# Atherogenic Index of Plasma Predicts the Onset and Progression of Cardio‐Renal‐Metabolic Multimorbidity: Evidence From a Nationwide Prospective Cohort Study

**DOI:** 10.1111/1753-0407.70227

**Published:** 2026-04-23

**Authors:** Jijie Jin, Fei Wang, Shengzhang Chen, Jiaxin Shao, Binyan Chen, Xiufang Lin

**Affiliations:** ^1^ The Center of Gerontology and Geriatrics National Clinical Research Center for Geriatrics West China Hospital, Sichuan University Chengdu China; ^2^ College of Nursing Wenzhou Medical University Wenzhou China

**Keywords:** atherogenic index of plasma, cardio‐renal‐metabolic, disease progression, lipid biomarkers, multimorbidity

## Abstract

**Background:**

Cardio‐renal‐metabolic (CRM) multimorbidity, defined by the co‐occurrence of cardiovascular disease (CVD), chronic kidney disease (CKD), and type 2 diabetes (T2DM), poses a growing burden for multimorbidity management. The atherogenic index of plasma (AIP), a lipid‐based marker of insulin resistance, may help predict CRM progression, but its prognostic value remains unclear.

**Methods:**

We used data from 5805 adults free of CRM at baseline in the China Health and Retirement Longitudinal Study. AIP was calculated as log_10_[triglyceride (TG)/high‐density lipoprotein cholesterol (HDL‐C)]. CRM onset was defined as incident CVD, T2DM, or CKD. Multistate models captured transitions from no disease to single, dual, and triple CRM states. Cox regression and time‐dependent receiver operating characteristic (ROC) curves were used to assess associations and predictive performance.

**Results:**

AIP showed a clear dose–response relationship with CRM progression. Each 1‐standard deviation (SD) increase in AIP was associated with higher risks of single (HR 1.17, 95% CI 1.13–1.22), dual (HR 1.31, 95% CI 1.21–1.41), and triple CRM (HR 1.57, 95% CI 1.27–1.94) diseases. Strong associations were observed for new‐onset T2DM and stroke, but not CKD. AIP yielded the highest discrimination for triple CRM (5‐year area under the curve [AUC] = 0.710). Multistate modeling showed each 1‐SD increase in AIP raised the hazard of transitioning from single to dual CRM by 14% and from dual to triple by 31%.

**Conclusions:**

AIP is an independent, simple, and low‐cost predictor of CRM onset and progression, with potential utility for early risk stratification and prevention.

## Highlights

Baseline atherogenic index of plasma (AIP) predicted the onset and stepwise progression of cardio‐renal‐metabolic (CRM) multimorbidity among 5805 participants from the China Health and Retirement Longitudinal Study (CHARLS) who were initially free of CRM conditions. Multistate models showed that each 1‐standard deviation (SD) increase in AIP was associated with higher hazards of transitions to single‐, dual‐, and triple‐CRM states, with the strongest associations observed for incident type 2 diabetes (T2DM) and stroke. AIP showed its best discrimination for 5‐year prediction of triple CRM (area under the curve [AUC] = 0.710), supporting its use as a low‐cost tool for risk stratification.

## Graphical abstract

Atherogenic index of plasma, calculated from triglycerides and HDL‐cholesterol, predicts the onset and stepwise progression of cardio‐renal‐metabolic multimorbidity. Multistate modeling in a nationwide prospective cohort shows higher AIP increases transitions to single, dual, and triple disease.

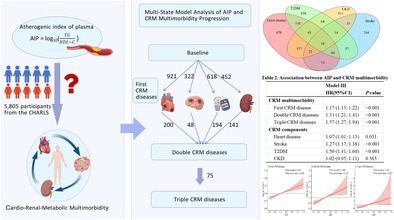

**Abbreviations**
AHA: American Heart Association
AIP: atherogenic index of plasma
AUC: area under the curve
BMI: body mass index
BUN: blood urea nitrogen
CHARLS: China Health and Retirement Longitudinal Study
CI: confidence interval
CKD: chronic kidney disease
CRM: cardio‐renal‐metabolic
CRP: C‐reactive protein
CVD: cardiovascular disease
HDL‐C: high‐density lipoprotein cholesterol
HR: hazard ratio
IR: insulin resistance
LDL‐C: low‐density lipoprotein cholesterol
RCS: restricted cubic splines
ROC: receiver operating characteristic
SD: standard deviation
T2DM: type 2 diabetes mellitus
TG: triglycerides
VIFs: variance inflation factors

## 1 Introduction

Cardio‐renal‐metabolic (CRM) disorders—encompassing type 2 diabetes mellitus (T2DM), cardiovascular disease (CVD), and chronic kidney disease (CKD)—are increasingly prevalent and represent a major global health challenge [1, 2]. These conditions frequently co‐occur, forming a multimorbidity cluster that is associated with heightened morbidity and mortality [3, 4]. In the United States, over 26% of adults live with at least one CRM condition, while the prevalence of two or more CRM disorders rose from 5.3% to 8.0% between 1999 and 2020 [5]. Coexisting conditions exert synergistic effects; for example, patients with both T2DM and CKD face a 10‐year mortality risk exceeding 30%, far higher than those with either disease alone [6, 7]. Consequently, international health guidelines now emphasize integrated preventive and therapeutic strategies that address common pathways across CRM conditions [8, 9].

CRM multimorbidity is driven by overlapping metabolic pathways, including insulin resistance, oxidative stress, endothelial dysfunction, and chronic inflammation [1, 9, 10]. These mechanisms contribute to a network of reciprocal organ damage, in which dysfunction in one system accelerates deterioration in the others [11, 12, 13]. Lipid metabolism plays a central role in this process. Elevated triglyceride‐rich lipoproteins and impaired high‐density lipoprotein cholesterol (HDL‐C) function promote systemic inflammation, vascular injury, and glucose intolerance, reinforcing the cycle of metabolic dysfunction [14, 15, 16].

The atherogenic index of plasma (AIP), calculated as log_10_[triglyceride (TG)/HDL‐C], has emerged as a robust marker of lipid‐driven insulin resistance and vascular risk [17, 18, 19]. Prior studies have shown its predictive value for individual diseases such as T2DM, CVD, and CKD [18, 20, 21]. However, existing evidence on AIP and CRM multimorbidity remains limited, as most prior studies have been cross‐sectional or focused on single‐disease outcomes rather than longitudinal multimorbidity trajectories. In particular, prospective nationwide evidence on the role of AIP in CRM multimorbidity progression is still lacking. To address this gap, we used longitudinal data from the China Health and Retirement Longitudinal Study (CHARLS) to investigate the prognostic utility of baseline AIP for CRM disease trajectories. Specifically, we aimed to: (1) quantify the association between AIP and initial CRM onset; (2) evaluate its role in accelerating transitions across CRM states; and (3) explore AIP's potential as a low‐cost, lipid‐based tool for early multimorbidity risk stratification in clinical settings.

## 2 Methods

### 2.1 Data Source and Study Population

The data for this study were derived from the CHARLS, a nationwide longitudinal cohort study targeting Chinese adults aged 45 years and above (http://charls.pku.edu.cn/). The initial national baseline survey was launched in 2011 using a multistage stratified sampling method, randomly selecting participants from 450 villages/communities across 150 counties/districts in 28 provinces. Participants were followed biennially. Detailed information regarding the study design and data collection procedures can be found in previous publications [22, 23, 24].

The participant selection process is illustrated in Figure S1. Starting with 16 931 participants from the 2011 baseline survey, 11 126 individuals were excluded for the following reasons: (1) 5514 lacked 2011 blood test data; (2) 188 had uncalculable AIP values; (3) 2754 had preexisting histories of heart disease, stroke, T2DM, or CKD at baseline; and (4) 2670 lacked follow‐up data for incident CRM diseases. Ultimately, 5805 participants meeting the inclusion criteria were enrolled.

The CHARLS study was conducted in accordance with the principles of the Declaration of Helsinki and received approval from the Peking University Institutional Review Board (IRB00001052‐11015). All participants provided written informed consent [24].

### 2.2 Definitions of Exposure

The AIP was defined as log_10_(TG/HDL‐C), both measured in mmol/L [18, 25]. Following standardized operating procedures established by the Chinese Center for Disease Control and Prevention, blood samples were collected by trained healthcare professionals and uniformly analyzed by the clinical laboratory at the Youanmen Clinical Trial Center, Capital Medical University, Beijing. TG and HDL‐C levels were measured via clinically standardized enzymatic colorimetric methods to ensure data quality [24].

### 2.3 Outcomes: CRM Multimorbidity States

The study outcome was the incidence of CRM conditions from 2011 to 2020. CRM multimorbidity was defined as the sequential development of CVD (heart disease or stroke), T2DM, and CKD during follow‐up [1, 4, 7, 26].

Outcomes were classified as:

- Single CRM disease: First diagnosis of any one of the following conditions during follow‐up: CVD (heart disease or stroke), T2DM, or CKD.
- Dual CRM disease: Presence of any two of the aforementioned conditions during follow‐up.
- Triple CRM disease: Development of all three conditions.

All participants were followed from baseline until the first event occurrence, loss to follow‐up, or study period conclusion. Detailed operational definitions of CRM multimorbidity are summarized in the Methods section of the Supporting Information.

### 2.4 Covariates

The covariates included age, sex, residential area (urban/rural), educational attainment (primary or below vs. secondary or above), marital status (married/partnered vs. other), current smoking, current alcohol use, body‐mass index (BMI), low‐density lipoprotein cholesterol (LDL‐C), blood urea nitrogen (BUN), platelet count, and C‐reactive protein (CRP).

### 2.5 Missing Data

Figure S2 illustrates the pattern of missingness. Because most variables had only low proportions of missing values, we used multiple imputation by chained equations (*m* = 5) under the missing‐at‐random assumption to minimize bias and preserve statistical power.

### 2.6 Statistical Analyses

Participants were stratified into four groups according to quartiles of AIP levels (Q1–Q4). Normally distributed continuous variables were expressed as the mean ± standard deviation (SD), with between‐group differences assessed using one‐way analysis of variance. Nonnormally distributed variables were presented as medians with interquartile ranges and were compared using the Kruskal–Wallis H test. Categorical variables were reported as frequencies (percentages), with group differences evaluated by the chi‐square test.

The associations between AIP (per 1‐SD increment) and the risk of CRM multimorbidity and its components (heart disease, stroke, T2DM, and CKD) were prospectively assessed using univariable and multivariable Cox proportional hazards regression models. Restricted cubic splines (RCS) were employed to visualize the dose–response relationship between AIP levels and the incidence of CRM multimorbidity. Additionally, to evaluate the time‐dependent predictive performance of AIP, time–dependent receiver operating characteristic (ROC) curve analysis was conducted to examine its predictive accuracy for incident single, dual, and triple CRM at 1‐, 3‐, and 5‐year time points.

Multistate models were used to characterize disease trajectories from a healthy state to progressive stages of CRM multimorbidity. Unlike traditional time‐to‐event analyses, this approach captures sequential transitions between disease states and provides a dynamic view of multimorbidity development [27, 28]. In this study, multistate modeling was used to assess the association between AIP and the progression of CRM multimorbidity. CRM progression was modeled as a three‐step process: (1) transition from no disease to single CRM condition; (2) progression to dual conditions; and (3) further advancement to triple CRM disease. In extended analyses, we constructed nine specific transition pathways according to the initial condition (heart disease, stroke, T2DM, or CKD) and evaluated how AIP influenced subsequent multimorbidity accumulation. We also compared the predictive performance of AIP across different disease initiation pathways to assess its stage‐specific utility. All models were adjusted for key demographic, behavioral, and biochemical covariates, including age, sex, residence, education, marital status, smoking, alcohol use, BMI, CRP, platelet count, LDL‐C, and BUN. This modeling strategy better reflects real‐world disease evolution and may inform more targeted, stage‐specific interventions for CRM multimorbidity.

To examine potential population heterogeneity in AIP‐CRM associations, subgroup analyses and interaction tests were performed by age (< 60 vs. ≥ 60 years), sex, BMI (< 24 vs. ≥ 24 kg/m^2^), residence (urban vs. rural), smoking status, and alcohol consumption status. For model robustness, variance inflation factors (VIFs) were calculated (Tables S1 and S2), with all covariates demonstrating VIFs < 5, indicating no substantial multicollinearity [29]. Additionally, sensitivity analyses using complete‐case datasets were conducted prior to multiple imputation to verify the consistency of the results (Tables S3 and S4). All statistical analyses were performed using R software (version 4.3.3), with two‐sided *p*‐values < 0.05 considered statistically significant.

## 3 Results

### 3.1 Baseline Characteristics

Among the 5805 participants (mean age 57.61 ± 8.54 years; 45.44% male), higher AIP quartiles were significantly associated with younger age and higher BMI, platelet counts, and CRP levels (all *p* < 0.05). Conversely, the proportion of current smokers decreased with increasing AIP quartiles (*p* = 0.002), and current drinking also differed significantly across quartiles (*p* < 0.001). The incidence of CRM multimorbidity rose markedly from the lowest to the highest AIP quartiles, particularly for T2DM, increasing from 6.34% in Q1 to 17.22% in Q4 (*p* < 0.001) (Table 1).

**TABLE 1:** Baseline characteristics of participants according to the atherogenic index of plasma.

Characteristics	AIP quartiles					
	Overall (*n* = 5805)	Quartile 1 (*n* = 1451)	Quartile 2 (*n* = 1450)	Quartile 3 (*n* = 1452)	Quartile 4 (*n* = 1452)	*p*
Age (years) mean ± SD	57.61 ± 8.54	58.32 ± 8.90	57.74 ± 8.63	57.48 ± 8.51	56.90 ± 8.04	< 0.001
Male, *n* (%)	2638 (45.44)	721 (49.69)	643 (44.34)	621 (42.77)	653 (44.97)	0.001
Married, *n* (%)	4997 (86.08)	1233 (84.98)	1228 (84.69)	1257 (86.57)	1279 (88.09)	0.030
Rural residence, *n* (%)	3988 (68.70)	1086 (74.84)	1018 (70.21)	969 (66.74)	915 (63.02)	< 0.001
Education level, *n* (%)						0.017
Elementary school or below	4019 (69.23)	1047 (72.16)	1005 (69.31)	998 (68.73)	969 (66.74)	
Middle school or above	1786 (30.77)	404 (27.84)	445 (30.69)	454 (31.27)	483 (33.26)	
BMI (kg/m^2^)^a^ median (Q_1_, Q_3_)	23.01 (20.80, 25.53)	21.61 (19.79, 23.71)	22.53 (20.55, 24.77)	23.58 (21.31, 26.14)	24.48 (22.25, 27.09)	0.044
TG (mg/dL)^a^ median (Q_1_, Q_3_)	102.66 (73.46, 148.68)	61.06 (52.22, 69.92)	86.73 (77.00, 99.12)	121.25 (105.32, 138.95)	199.12 (162.84, 257.54)	< 0.001
BUN (mg/dL) mean ± SD	15.65 ± 4.39	16.51 ± 4.73	15.67 ± 4.32	15.16 ± 4.23	15.27 ± 4.13	< 0.001
CRP (mg/dL)^a^ median (Q_1_, Q_3_)	0.93 (0.51, 1.92)	0.73 (0.43, 1.55)	0.82 (0.49, 1.74)	0.98 (0.55, 1.98)	1.21 (0.66, 2.33)	< 0.001
Blood platelet (×10^9^/L) mean ± SD	211.38 ± 72.77	205.29 ± 68.29	212.27 ± 75.98	212.66 ± 72.10	215.31 ± 74.21	0.002
HDL‐C (mg/dL) mean ± SD	51.69 ± 15.24	67.26 ± 14.08	54.69 ± 10.06	47.51 ± 8.82	37.30 ± 8.72	< 0.001
LDL‐C (mg/dL) mean ± SD	116.13 ± 34.48	111.65 ± 29.54	119.36 ± 32.23	123.02 ± 33.50	110.50 ± 40.18	< 0.001
Current smoker, *n* (%)	1766 (30.42)	497 (34.25)	433 (29.86)	410 (28.24)	426 (29.34)	0.002
Current drinker, *n* (%)	2383 (41.05)	666 (45.90)	576 (39.72)	561 (38.64)	580 (39.94)	< 0.001
Single CRM disease, *n* (%)	2313 (39.84)	485 (33.43)	543 (37.45)	624 (42.98)	661 (45.52)	< 0.001
Dual CRM disease, *n* (%)	583 (10.04)	91 (6.27)	131 (9.03)	167 (11.50)	194 (13.36)	< 0.001
Triple CRM disease, *n* (%)	75 (1.29)	6 (0.41)	21 (1.45)	19 (1.31)	29 (2.00)	< 0.001
First‐onset heart disease, *n* (%)	921 (15.87)	213 (14.68)	221 (15.24)	270 (18.60)	217 (14.94)	0.012
First‐onset stroke, *n* (%)	322 (5.55)	66 (4.55)	73 (5.03)	92 (6.34)	91 (6.27)	< 0.001
First‐onset T2DM, *n* (%)	618 (10.65)	92 (6.34)	127 (8.76)	149 (10.26)	250 (17.22)	< 0.001
First‐onset CKD, *n* (%)	452 (7.79)	114 (7.86)	122 (8.41)	113 (7.78)	103 (7.09)	0.620

*Note:* Continuous variables were presented as mean ± SD for normally distributed data and as median (interquartile range) for nonnormally distributed data. Differences among groups were assessed using one‐way analysis of variance (ANOVA) or the Kruskal–Wallis test, as appropriate. Categorical variables were presented as counts (percentages) and compared using the chi‐square test or Fisher's exact test when expected cell counts were < 10.

Abbreviations: AIP, atherogenic index of plasma; BMI, body mass index; BUN, blood urea nitrogen; CKD, chronic kidney disease; CRM, cardio‐renal‐metabolic; CRP, C‐reactive protein; HDL‐C, high‐density lipoprotein cholesterol; LDL‐C, low‐density lipoprotein cholesterol; SD, standard deviation; TG, triglycerides.

^a^ Presented as median (interquartile range).

### 3.2 Relationship Between AIP and CRM Multimorbidity

In fully adjusted Cox models, each 1‐SD increase in AIP was associated with a 17% higher risk of single CRM disease (HR 1.17, 95% CI: 1.13–1.22), 31% higher risk for dual disease (HR 1.31, 95% CI: 1.21–1.41), and 57% higher risk for triple disease (HR 1.57, 95% CI: 1.27–1.94). These associations remained statistically significant in the crude and age‐ and sex‐adjusted models and were slightly strengthened after full adjustment (Table 2).

**TABLE 2:** Association between AIP and CRM multimorbidity.

	Crude model		Model II		Model III	
	HR (95% CI)	*p*	HR (95% CI)	*p*	HR (95% CI)	*p*
CRM multimorbidity						
Single CRM disease	1.16 (1.12, 1.20)	< 0.001	1.16 (1.12, 1.21)	< 0.001	1.17 (1.13, 1.22)	< 0.001
Dual CRM diseases	1.25 (1.16, 1.35)	< 0.001	1.26 (1.17, 1.36)	< 0.001	1.31 (1.21, 1.41)	< 0.001
Triple CRM diseases	1.43 (1.18, 1.74)	< 0.001	1.47 (1.21, 1.79)	< 0.001	1.57 (1.27, 1.94)	< 0.001
CRM components						
Heart disease	1.05 (0.99, 1.11)	0.123	1.05 (0.99, 1.11)	0.083	1.07 (1.01, 1.13)	0.031
Stroke	1.21 (1.12, 1.31)	< 0.001	1.24 (1.14, 1.34)	< 0.001	1.27 (1.17, 1.38)	< 0.001
T2DM	1.44 (1.36, 1.53)	< 0.001	1.44 (1.36, 1.53)	< 0.001	1.50 (1.41, 1.60)	< 0.001
CKD	1.03 (0.92, 1.08)	0.484	1.01 (0.94, 1.09)	0.781	1.02 (0.95, 1.11)	0.565

*Note:* Model I: Crude model. Model II: Adjusted for age, sex. Model III: adjusted for age, sex, residence, education level, marital status, smoking status, drinking status, BMI, CRP, Blood platelet, LDL‐C, BUN.

**FIGURE 1:**
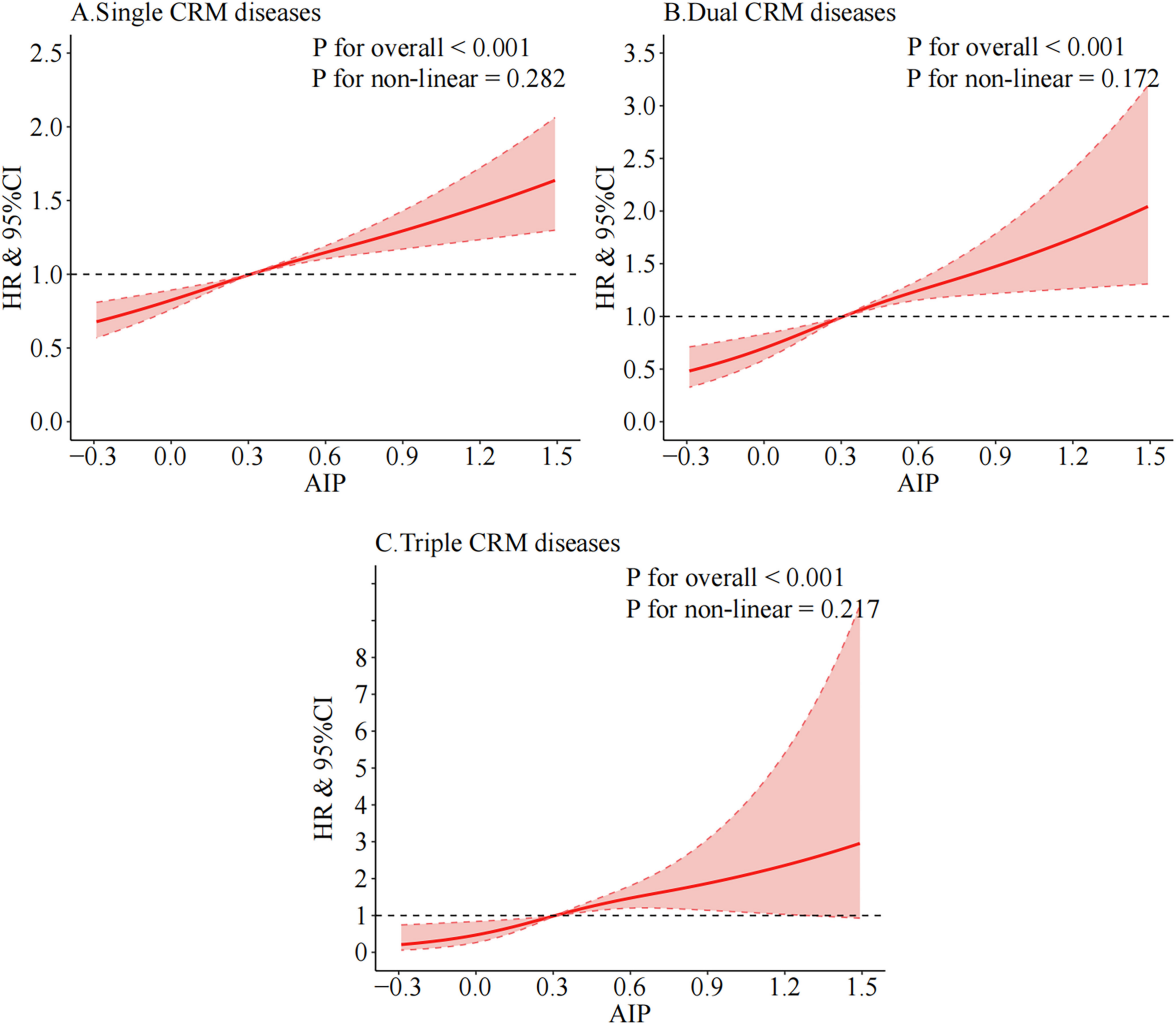
Cubic model of the associations between different CRM multimorbidity stages and AIP: (A) single CRM disease, (B) dual CRM disease, and (C) triple CRM disease. AIP, atherogenic index of plasma; CRM, cardio‐renal‐metabolic.

Time‐dependent ROC analysis showed moderate discriminatory performance of AIP, with the highest 5‐year area under the curve (AUC) observed for triple CRM disease (AUC = 0.710; Figure S3), suggesting stronger predictive utility as multimorbidity complexity increases. RCS analyses demonstrated linear dose–response relationships between AIP and the risk of CRM outcomes (*p* for non‐linear > 0.05; Figure 1).

Notably, among individual CRM components, AIP was most strongly associated with incident T2DM (HR 1.50, 95% CI: 1.41–1.60), followed by stroke (HR 1.27, 95% CI: 1.17–1.38) and heart disease (HR 1.07, 95% CI: 1.01–1.13), whereas no significant association was observed with CKD (HR 1.02, 95% CI: 0.95–1.11; *p* = 0.565) (Table 2).

**FIGURE 2:**
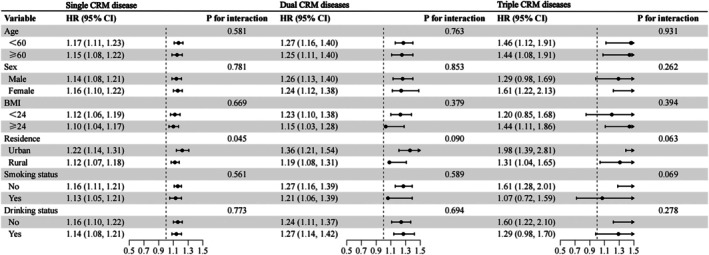
Subgroup analysis forest plot of the comorbidity correlation between AIP and different CRM multimorbidity stages. AIP, atherogenic index of plasma; BMI, body mass index; CRM, cardio‐renal‐metabolic.

Subgroup analyses showed no interaction by age, sex, BMI, smoking, or alcohol use (all *p* for interaction > 0.05). A significant interaction by residence was observed for single CRM disease: the association between AIP and single CRM disease was stronger in urban residents (HR 1.22, 95% CI: 1.14–1.31) than in rural residents (HR 1.12, 95% CI: 1.07–1.18; *p* for interaction = 0.045) (Figure 2).

### 3.3 Relationship Between AIP and CRM Multimorbidity Trajectories

Among the 5805 participants free of CRM disease at baseline, 2313 (39.84%) developed at least one CRM disease during follow‐up; among these incident cases, 583 (25.20%) progressed to dual CRM disease and 75 (3.24%) progressed to triple CRM disease. In multistate models (Table 3, Model 1), each 1‐SD increase in AIP was associated with a 17% higher hazard of transitioning from healthy to single CRM disease (HR 1.17, 95% CI: 1.13–1.22), a 14% increase from single to dual disease (HR 1.14, 95% CI: 1.05–1.23), and a 31% increase from dual to triple disease (HR 1.31, 95% CI: 1.02–1.67). In subtype‐specific trajectories, AIP showed heterogeneous associations across CRM components. Each 1‐SD increase was linked to incident T2DM (HR 1.52, 95% CI: 1.42–1.63), stroke (HR 1.23, 95% CI: 1.11–1.37), and heart disease (HR 1.06, 95% CI: 1.01–1.14), but not to CKD (HR 0.98, 95% CI: 0.89–1.08, *p* = 0.668). For transitions from single to dual CRM disease, AIP remained predictive when the initial condition was heart disease (HR 1.16, 95% CI: 1.05–1.28), stroke (HR 1.28, 95% CI: 1.08–1.52), or CKD (HR 1.13, 95% CI: 1.01–1.27), but not T2DM (HR 0.93, 95% CI: 0.84–1.03, *p* = 0.148). For transitions from dual to triple CRM disease, elevated AIP again increased risk (HR 1.31, 95% CI: 1.02–1.68; *p* = 0.035) (Table 3 Model 2).

**TABLE 3:** Association between AIP and the trajectory of CRM multimorbidity via multi‐state model.

Transition	HR (95% CI)	*p*
Model 1: Baseline → single CRM disease → dual CRM diseases → triple CRM diseases		
Baseline → single CRM disease	1.17 (1.13, 1.22)	< 0.001
Single CRM disease → dual CRM diseases	1.14 (1.05, 1.23)	0.002
Dual CRM diseases → triple CRM diseases	1.31 (1.02, 1.67)	0.034
Model 2: Baseline → single CRM disease subtypes (heart disease, stroke, T2DM, and CKD) → dual CRM diseases → triple CRM diseases		
Baseline → heart disease	1.06 (1.01, 1.14)	0.032
Baseline → stroke	1.23 (1.11, 1.37)	< 0.001
Baseline → T2DM	1.52 (1.42, 1.63)	< 0.001
Baseline → CKD	0.98 (0.89, 1.08)	0.668
Heart disease→ dual CRM diseases	1.16 (1.05, 1.28)	0.005
Stroke → dual CRM diseases	1.28 (1.08, 1.52)	0.004
T2DM → dual CRM diseases	0.93 (0.84, 1.03)	0.148
CKD → dual CRM diseases	1.13 (1.01, 1.27)	0.037
Dual CRM diseases → Triple CRM diseases	1.31 (1.02, 1.68)	0.035

*Note:* Adjusted model adjusted for age, sex, residence, education level, marital status, smoking status, drinking status, BMI, CRP, blood platelet, LDL‐C, BUN.

## 4 Discussion

In this large prospective Chinese cohort, higher AIP was significantly associated with an increased risk of both CRM disease onset and multimorbidity progression. A clear dose–response relationship was observed: each 1‐SD increase in AIP was associated with a 17% higher risk of developing a single CRM disease, a 31% higher risk of dual CRM diseases, and a 57% higher risk of triple CRM diseases. Furthermore, elevated AIP not only predicted the incidence of single conditions but also accelerated disease progression, increasing the transition risk from single to dual CRM diseases by 14% and from dual to triple CRM diseases by 31%. The strongest predictive power was observed in advanced multimorbidity, particularly for triple‐disease states. Notably, the strongest individual‐level association was observed between AIP and incident type 2 diabetes, consistent with its mechanistic link to insulin resistance and lipid‐driven metabolic dysfunction. Given the central role of T2DM in initiating cardio‐metabolic comorbid chains, this finding reinforces the utility of AIP not only as a diabetes risk marker but also as a trigger‐point indicator for CRM multimorbidity progression.

Mechanistically, the stronger predictive effect of AIP across transitions from single disease to multimorbidity may reflect the cumulative impact of lipid‐driven metabolic dysfunction over time [19, 30]. AIP, defined as log_10_(TG/HDL‐C), captures both elevated triglycerides and reduced HDL‐C levels [25, 31]. In conditions of high AIP, triglyceride‐rich lipoproteins such as very‐low‐density lipoprotein (VLDL) and their remnants increase vascular permeability, activate inflammatory pathways including nuclear factor kappa B (NF‐κB) signaling, and promote oxidative stress and atherosclerotic plaque formation [32]. Simultaneously, low HDL‐C impairs reverse cholesterol transport and diminishes HDL's anti‐inflammatory and antioxidant functions [33]. This combined dyslipidemic imbalance contributes to lipotoxicity, insulin resistance, endothelial dysfunction, and multi‐organ damage [34], thereby facilitating both the onset of individual CRM conditions and their progression to multimorbidity. In early disease stages, these metabolic disturbances may contribute primarily to the development of a single CRM condition. However, as disease burden accumulates, persistent insulin resistance and chronic low‐grade systemic inflammation may further amplify cross‐organ interactions [20, 35], which may help explain the stronger predictive effect of AIP in later transitions from single disease to dual and triple CRM multimorbidity. The relatively weaker association observed between AIP and CKD may reflect the heterogeneous and multifactorial nature of renal pathogenesis, which is not solely driven by lipid abnormalities but may also involve other non‐lipid‐related pathways, such as hemodynamic stress, microvascular injury, and fibrotic remodeling [36, 37]. In addition, malnutrition‐inflammation status in some patients may further complicate the relationship between lipid‐related markers and renal outcomes [38]. Collectively, AIP serves as a comprehensive biomarker of dyslipidemia‐induced inflammation and oxidative stress, providing a biological basis for its role in CRM disease progression.

This study advances current knowledge by applying a longitudinal, multistate modeling framework to assess CRM risk dynamically. Most previous AIP studies focused on single diseases or cross‐sectional associations. Here, we tracked transitions across four stages—healthy, single, dual, and triple CRM multimorbidity—and demonstrated that AIP's influence intensifies over time. To our knowledge, this is the first study to capture the full CRM trajectory using AIP in an Asian population.

Importantly, AIP's predictive ability is not static. It varies across different stages of CRM disease. In early phases, such as the onset of a single CRM condition, AIP demonstrates good short‐term predictive performance. This may reflect its high sensitivity to early lipid and metabolic disturbances, as previously reported by Andraschko et al. [19]. However, as the disease burden increases, its predictive accuracy gradually weakens. In more advanced stages, combining AIP with other biomarkers—such as glycemic or inflammatory indicators—may improve risk stratification. In contrast, AIP retains strong capacity to predict triple CRM disease. This finding is consistent with the results reported by Tang et al. [34]. It likely reflects the cumulative metabolic dysregulation seen in later stages of multimorbidity, where AIP may capture global metabolic imbalance more effectively. Notably, although AIP is a well‐established predictor of T2DM onset [21, 39], its ability to predict progression to dual CRM disease in patients with existing diabetes was markedly reduced. This suggests a stage‐specific shift in dominant pathophysiological mechanisms. In early disease, lipid metabolism abnormalities appear to play a central role. In later phases, glucose dysregulation and chronic systemic inflammation may become the primary drivers of disease progression [40, 41]. This stage‐specific effect was particularly evident in individuals with established T2DM, where AIP lost its predictive value for further disease accumulation. These findings suggest that combining AIP with complementary biomarkers (e.g., glycemic or inflammatory markers) may enhance predictive accuracy across CRM stages.

These findings support a dual clinical role for AIP. First, it can serve as a screening tool to identify high‐risk individuals at early stages. Second, it may help monitor disease evolution and inform timely interventions. Given that AIP is derived from routine lipid panels [42], it offers a simple, scalable, and cost‐effective biomarker for use in both clinical practice and public health. Individuals with elevated AIP may benefit from early interventions, including lifestyle modifications aimed at reducing triglycerides and improving HDL levels [43, 44]. When lifestyle changes are insufficient, AIP could guide therapeutic decisions—prompting clinicians to consider non‐statin treatments (e.g., fibrates, omega‐3 fatty acids, niacin) targeting triglyceride‐rich lipoproteins and HDL dysfunction [45, 46]. Longitudinal monitoring of AIP could help guide timely therapeutic adjustments as patients move from single to more complex CRM conditions. Subgroup analyses indicated the risk of triple CRM disease associated with high AIP was more pronounced among women—possibly reflecting loss of estrogen protection post‐menopause—and individuals with obesity, where chronic inflammation and insulin resistance may amplify lipid toxicity [2, 47, 48]. Such insights underscore the need for tailored, sex‐ and weight‐specific prevention strategies based on AIP.

Beyond individual care, the integration of AIP into community‐based screening programs could have significant public health value. Its simplicity and wide availability make it suitable for early detection efforts in primary care. These findings suggest that AIP‐based risk stratification may support scalable, low‐cost strategies to address the growing burden of CRM multimorbidity, particularly in aging and resource‐constrained populations.

Strengths of our study include its large sample size, prospective design, and use of multistate modeling to reflect real‐world disease dynamics. Nonetheless, several limitations should be acknowledged. First, residual confounding cannot be excluded because of the observational nature of the study. Second, our study focused on CRM multimorbidity as the coexistence and progression of CVD, T2DM, and CKD, rather than on the American Heart Association (AHA)‐defined cardiovascular‐kidney‐metabolic (CKM) syndrome [49]. Therefore, direct comparisons with CKM‐based studies should be interpreted cautiously, and this difference in disease definition may have affected diagnostic precision. Third, death was not incorporated as a competing event or absorbing state in the multistate model because mortality data were unavailable in the analytic dataset used for this study. As a result, participants who died during follow‐up were effectively censored, which may have affected transition estimates if mortality was associated with baseline AIP or underlying CRM progression risk. Finally, AIP was measured only at baseline; the lack of serial lipid measurements precludes assessment of how changes in AIP over time influence disease progression risk. Future studies should investigate dynamic AIP trajectories and, where possible, incorporate mortality into multistate frameworks to provide a more complete characterization of CRM progression.

## 5 Conclusion

In this nationwide prospective cohort, the AIP independently predicted both the onset and progression of CRM multimorbidity. These findings support the clinical utility of AIP as a simple, cost‐effective biomarker for early risk identification. Its integration into routine care and population‐level screening strategies may help enable timely, targeted interventions to alleviate the rising burden of CRM multimorbidity in aging populations.

## Author Contributions

Jijie Jin and Fei Wang wrote the manuscript, Jijie Jin visualized the data, Shengzhang Chen verified the data, Fei Wang and Binyan Chen collected the data, Jiaxin Shao operated the software, Xiufang Lin conceived the methodology and proposed the project concept. All authors reviewed the manuscript. All the authors contributed to the article and approved the submitted version.

## Funding

The authors have nothing to report.

## Ethics Statement

The entire study process adhered to the Declaration of Helsinki, and the study results were reported following the STROBE guidelines. The protocol for the CHARLS cohort was authorized by the Ethics Review Committee of Peking University (IRB00001052‐11015).

## Consent

All participants provided written informed consent at the time of participation.

## Conflicts of Interest

The authors declare no conflicts of interest.

## Supporting information


**Table S1:** Variance inflation factors of covariates in the adjusted model for the association between AIP and CRM multimorbidity.
**Table S2:** Variance inflation factors of covariates in the adjusted model for the association between AIP and CRM components.
**Table S3:** Association between AIP and CRM multimorbidity in participants with complete data.
**Table S4:**. Association between AIP and CRM multimorbidity trajectory using multi‐state model in participants with complete data.
**Figure S1:** Selection process of the study population.
**Figure S2:** Distribution of variables with missing data.
**Figure S3:** Predictive power of AIP for CRM multimorbidity. The area under the receiver operating characteristic curve of (A) Single CRM disease, (B) Dual CRM diseases, and (C) Triple CRM diseases.

## Data Availability

The data that support the findings of this study are available from the China Health and Retirement Longitudinal Study (CHARLS) website, subject to registration and application process. Further details can be found at http://www.charls.pku.edu.cn/en.
