# Supplementary material for: Atherogenic Index of Plasma Predicts the Onset and Progression of Cardio‐Renal‐Metabolic Multimorbidity: Evidence From a Nationwide Prospective Cohort Study
Source: J Diabetes. 2026 Apr 23;18(4):e70227. doi: 10.1111/1753-0407.70227 (PMC13104600; doi:10.1111/1753-0407.70227)
Supplement: Supplementary file 1 — Table S1: Variance inflation factors of covariates in the adjusted model for the association between AIP and CRM multimorbidity. Table S2: Variance inflation factors of covariates in the adjusted model for the association between AIP and CRM components. Table S3: Association between AIP and CRM multimorbidity in participants with complete data. Table S4:. Association between AIP and CRM multimorbidity trajectory using multi‐state model in participants with complete data. Figure S1: Selection process of the study population. Figure S2: Distribution of variables with missing data. Figure S3: Predictive power of AIP for CRM multimorbidity. The area under the receiver operating characteristic curve of (A) Single CRM disease, (B) Dual CRM diseases, and (C) Triple CRM diseases. [file JDB-18-e70227-s001.docx]

**Supplemental materials**

**Definition of specific subtypes of CRM disease**

**Supplemental Table 1.** Variance inflation factors of covariates in the adjusted model for the association between AIP and CRM Multimorbidity

**Supplemental Table 2.** Variance inflation factors of covariates in the adjusted model for the association between AIP and CRM components

**Supplemental Table 3.** Association between AIP and CRM Multimorbidity in Participants with Complete Data

**Supplemental Table 4.** Association between AIP and CRM multimorbidity trajectory using multi-state model in participants with complete data.

**Supplementary Figure 1.** Selection process of the study population.

**Supplementary Figure 2.** Distribution of variables with missing data.

**Supplementary Figure 3.** Predictive power of AIP for CRM multimorbidity. The area under the receiver operating characteristic curve of A Single CRM disease, B Dual CRM diseases, and Triple CRM diseases.

**Definition of specific subtypes of CRM disease**

Since previous studies have shown that self-reported chronic diseases are reasonably reliable and this approach has been widely used in studies based on the China Health and Retirement Longitudinal Study (CHARLS), we used participants’ self-reported physician diagnoses to ascertain disease events(1-3). This study focused on specific subtypes of cardio-renal-metabolic (CRM) disease, including heart disease, stroke, type 2 diabetes mellitus (T2DM), and chronic kidney disease (CKD). For each disease, participants were followed individually from the 2011 baseline survey until the first occurrence of that disease or the last available survey in 2020, whichever came first.

The timing of each disease event was defined according to the participant’s self-reported time of first diagnosis or first awareness of that condition, based on the corresponding CHARLS questionnaire item, rather than being assigned only according to the wave of the follow-up interview. Accordingly, in the multistate analysis, the transition from no CRM disease to single CRM disease was dated at the first reported onset of any CRM component; the transition from single to dual CRM disease was dated at the first reported onset of the second CRM component; and the transition from dual to triple CRM disease was dated at the first reported onset of the third CRM component.

Heart Disease

Heart disease was determined based on the participant's response to the standardized question in the questionnaire: "Has the doctor ever told you that you have been diagnosed with a heart attack, coronary heart disease, angina, heart failure, or other heart problems?" A heart disease event was considered to have occurred if the answer was "yes." The time of the event was determined by the following questions: "When was the first time you diagnosed or learned that you had heart disease (such as myocardial infarction, coronary heart disease, angina, congestive heart failure, and other heart diseases)?" and "When was your last heart disease (such as myocardial infarction, coronary heart disease, angina, congestive heart failure, and other heart diseases)?"(4).

Stroke

The definition of stroke events was based on the participant's answer to the question "Has your doctor ever told you that you have been diagnosed with stroke?" If the answer was affirmative, it was considered a stroke event. The time of the event was determined by the following questions: "When did you first diagnose or learn about your stroke?" and "When was your last stroke?"(5).

T2DM

T2DM was determined based on the participant's answer to the question "Has your doctor told you that you have been diagnosed with type 2 diabetes?" in the questionnaire. If the answer was "yes", it was considered confirmed diabetes. The time of first onset was determined by the participant's answer to the question "When did you first diagnose or learn that you had diabetes?"(6).

CKD

CKD was defined based on participants’ responses to the question “Have you been told by your doctor that you have been diagnosed with kidney disease?” If yes, a CKD event was considered to have occurred. The time of onset was determined by the question "When did you first diagnose or learn that you had CKD?"(7).

**Reference**

1. Gao K, Cao L-F, Ma W-Z, Gao Y-J, Luo M-S, Zhu J, et al. Association between sarcopenia and cardiovascular disease among middle-aged and older adults: Findings from the China health and retirement longitudinal study. EClinicalMedicine. 2022;44:101264.

2. Najafi F, Moradinazar M, Hamzeh B, Rezaeian S. The reliability of self-reporting chronic diseases: how reliable is the result of population-based cohort studies. J Prev Med Hyg. 2019;60(4):E349-E53.

3. Song Y, Zhu C, Shi B, Song C, Cui K, Chang Zg, et al. Social isolation, loneliness, and incident type 2 diabetes mellitus: results from two large prospective cohorts in Europe and East Asia and Mendelian randomization. EClinicalMedicine. 2023;64:102236.

4. Tang X, Zhang K, He R. The association of triglyceride-glucose and triglyceride-glucose related indices with the risk of heart disease in a national. Cardiovasc Diabetol. 2025;24(1):54.

5. Huo G, Tang Y, Liu Z, Cao J, Yao Z, Zhou D. Association between C-reactive protein-triglyceride glucose index and stroke risk in different glycemic status: insights from the China Health and Retirement Longitudinal Study (CHARLS). Cardiovasc Diabetol. 2025;24(1):142.

6. Cui C, Song J, Zhang L, Han N, Xu W, Sheng C, et al. The additive effect of the stress hyperglycemia ratio on type 2 diabetes: a population-based cohort study. Cardiovasc Diabetol. 2025;24(1):5.

7. Cao Y, Tang M, Zhao J, Yin L. Association of combined left and right handgrip strength with new-onset chronic kidney disease in middle-aged and older adults: a nationwide multicenter cohort study. BMC Public Health. 2025;25(1):988.

**Supplemental Table 1. Variance inflation factors of covariates in the adjusted model for the association between AIP and CRM Multimorbidity**

| Variable | Single CRM disease | Dual CRM diseases | Triple CRM diseases |
| --- | --- | --- | --- |
|  | VIF | VIF | VIF |
| AIP(per SD) | 1.034998 | 1.037279 | 1.082174 |
| Age | 1.179619 | 1.165449 | 1.181888 |
| Sex | 1.996879 | 1.962232 | 1.915852 |
| Residence | 1.052904 | 1.056165 | 1.093114 |
| Education level | 1.240711 | 1.237178 | 1.241398 |
| Marital status | 1.051744 | 1.04956 | 1.056392 |
| Smoking status | 1.576771 | 1.517953 | 1.424541 |
| Drinking status | 1.412698 | 1.413068 | 1.451989 |
| BMI | 1.008615 | 1.012092 | 1.140220 |
| CRP | 1.011318 | 1.010873 | 1.027249 |
| BUN | 1.056831 | 1.050568 | 1.080462 |
| LDL-C | 1.064163 | 1.064016 | 1.110803 |
| Blood platelet | 1.043106 | 1.041514 | 1.071664 |

Abbreviations: BMI, body mass index; BUN, Blood urea nitrogen; CRP, C-reactive protein; LDL-C, low density lipoprotein; AIP, atherogenic index of plasma; VIF, variance inflation factor.

**Supplemental Table 2. Variance inflation factors of covariates in the adjusted model for the association between AIP and CRM components**

| Variable | Heart disease | Stroke | T2DM | CKD |
| --- | --- | --- | --- | --- |
|  | VIF | VIF | VIF | VIF |
| AIP（per SD) | 1.026096 | 1.041908 | 1.071068 | 1.034922 |
| Age | 1.191790 | 1.162204 | 1.162378 | 1.156628 |
| Sex | 1.995291 | 1.960046 | 1.960984 | 1.891035 |
| Residence | 1.063975 | 1.048531 | 1.056411 | 1.051958 |
| Education level | 1.259399 | 1.213156 | 1.238190 | 1.212202 |
| Marital status | 1.056280 | 1.065183 | 1.048913 | 1.031659 |
| Smoking status | 1.572498 | 1.537337 | 1.527756 | 1.488933 |
| Drinking status | 1.392271 | 1.412431 | 1.416334 | 1.381152 |
| BMI | 1.011373 | 1.004237 | 1.012166 | 1.013772 |
| CRP | 1.009753 | 1.012881 | 1.013649 | 1.009519 |
| BUN | 1.062587 | 1.048146 | 1.052118 | 1.062002 |
| LDL-C | 1.051780 | 1.061455 | 1.105072 | 1.051063 |
| Blood platelet | 1.043756 | 1.037914 | 1.042058 | 1.039801 |

Abbreviations: BMI, body mass index; BUN, Blood urea nitrogen; CRP, C-reactive protein; LDL-C, low density lipoprotein; AIP, atherogenic index of plasma; VIF, variance inflation factor.

**Supplemental Table 3.** Association between AIP and CRM Multimorbidity in Participants with Complete Data

|  | **Crude Model** |  | **Adjusted model** |  |
| --- | --- | --- | --- | --- |
|  | **HR (95% CI)** | ***P* value** | **HR (95% CI)** | ***P* value** |
| **CRM multimorbidity** |  |  |  |  |
| Single CRM disease | 1.18 (1.13, 1.23) | <0.001 | 1.20 (1.14, 1.25) | <0.001 |
| Dual CRM diseases | 1.30 (1.19, 1.40) | <0.001 | 1.35 (1.24, 1.48) | <0.001 |
| Triple CRM diseases | 1.50 (1.21, 1.86) | <0.001 | 1.66 (1.32, 2.10) | <0.001 |
| **CRM components** |  |  |  |  |
| Heart disease | 1.06 (1.01, 1.13) | 0.044 | 1.07 (1.00, 1.14) | 0.049 |
| Stroke | 1.26 (1.15, 1.37) | <0.001 | 1.32 (1.20, 1.45) | <0.001 |
| T2DM | 1.47 (1.38, 1.57) | <0.001 | 1.54 (1.43, 1.65) | <0.001 |
| CKD | 1.03 (0.94, 1.12) | 0.560 | 1.07 (0.97, 1.17) | 0.171 |

The crude model did not account for covariates

The adjusted model was adjusted for Age, Sex, Residence, Education level, Marital status, Smoking status, Drinking status, BMI, CRP, Blood platelet, LDL-C, BUN

**Supplemental Table 4.** Association between AIP and CRM multimorbidity trajectory via multi-state model in participants with complete data.

| **Transition** | **HR (95% CI)** | ***P* value** |
| --- | --- | --- |
| **Baseline → Single CRM disease → Dual CRM diseases → Triple CRM diseases** | | |
| Baseline → Single CRM disease | 1.20 (1.14, 1.25) | <0.001 |
| Single CRM disease → Dual CRM diseases | 1.14 (1.05, 1.25) | 0.003 |
| Dual CRM diseases → Triple CRM diseases | 1.36 (1.04, 1.79) | 0.026 |

Adjusted model adjusted for Age, Sex , Residence, Education level, Marital status, Smoking status, Drinking status, BMI, CRP, Blood platelet, LDL-c, BUN

**
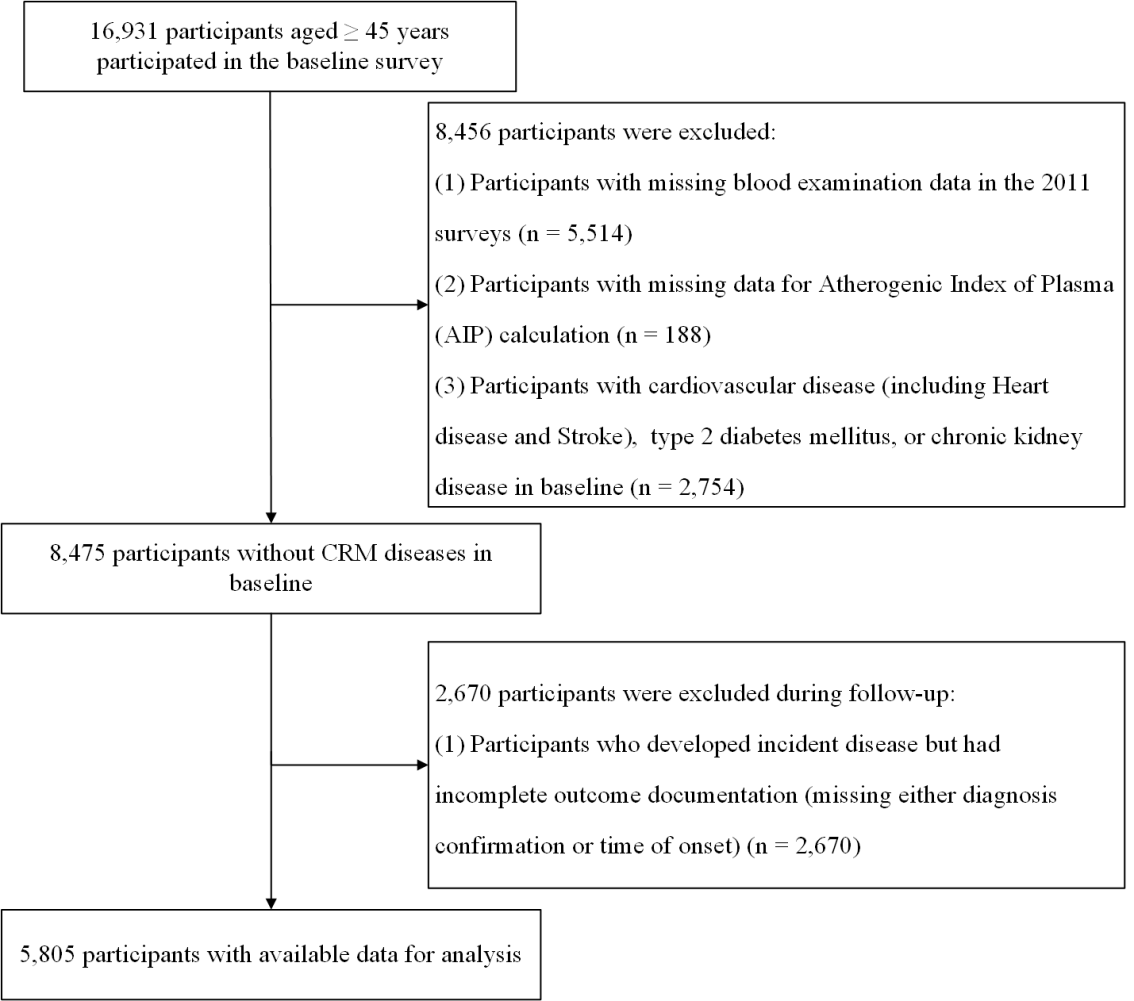
**

**Supplementary Figure 1.** Selection process of the study population.

**
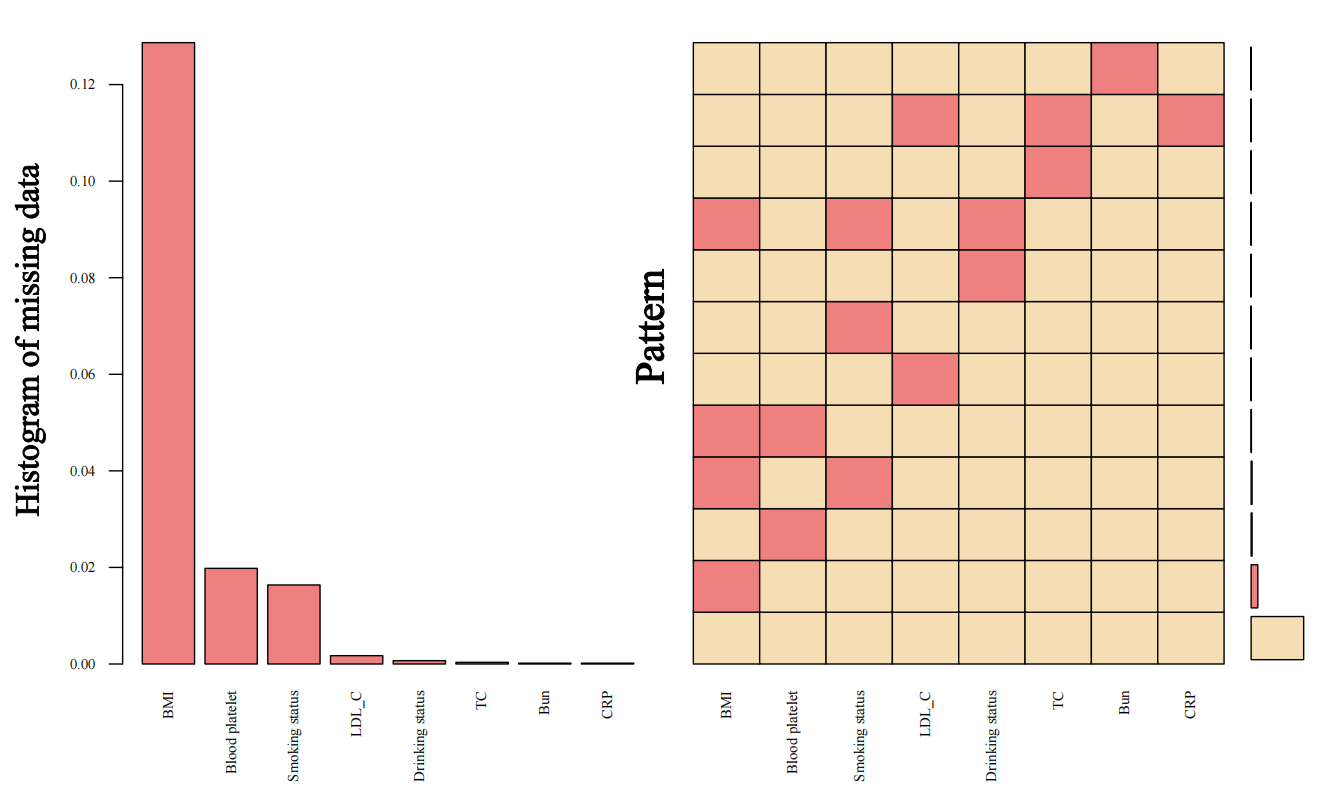
**

**Supplementary Figure 2.** Distribution of variables with missing data.

Abbreviations: BMI, body mass index; LDL-C, low density lipoprotein; TC, total cholesterol; BUN, Blood urea nitrogen; CRP, C-reactive protein

**
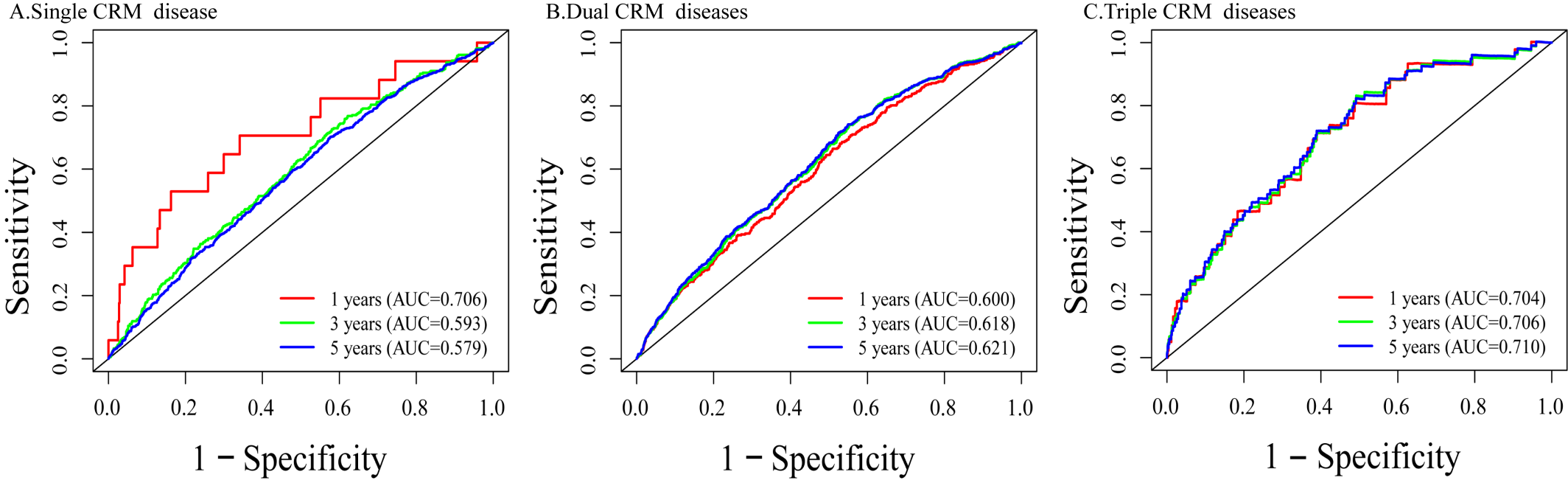
**

**Supplementary Figure 3.** Predictive power of AIP for CRM multimorbidity. The area under the receiver operating characteristic curve of (A) Single CRM disease, (B) Dual CRM diseases, and (C) Triple CRM diseases.
